# Optimizing Physical Activity and Decreasing Care-Resistant Behaviors Among Nursing Home Residents With Dementia

**DOI:** 10.1093/geroni/igaa057.2152

**Published:** 2020-12-16

**Authors:** Elizabeth Galik, Barbara Resnick, Erin Vigne, Sarah Holmes

**Affiliations:** 1 University of Maryland School of Nursing, Ellicott City, Maryland, United States; 2 University of Maryland School of Nursing, Baltimore, Maryland, United States; 3 University of Maryland School of Pharmacy, Baltimore, Maryland, United States

## Abstract

The purpose of this study was to test the effectiveness of the Function and Behavior Focused Care (FBFC) intervention on function, physical activity and behavioral symptoms among nursing home residents with dementia. This study was a clustered, randomized controlled trial with a repeated measures design in 12 nursing homes. The participants (N=336) were 82.6 (SD=10.1) years of age, mostly female and were moderate to severely cognitively impaired (MMSE of 7.8, SD=5.1). There were a statistically significant improvements in time spent in light, moderate and overall physical activity based on actigraphy and a decrease in resistiveness to care at 4 months among participants in the treatment group. There was no change in mood, agitation, and the use of psychotropic medications. This study provides some support for the use of the FBFC intervention to increase time spent in physical activity and decrease resistive behaviors during care among nursing home residents with dementia. Part of a symposium sponsored by Nursing Care of Older Adults Interest Group.

